# Psychometric properties of the short-form UCLA Loneliness Scale (ULS-8) among Chinese adolescents

**DOI:** 10.1097/MD.0000000000012373

**Published:** 2018-09-21

**Authors:** Shurong Xu, Dan Qiu, Jessica Hahne, Mei Zhao, Mi Hu

**Affiliations:** a Department of Social Medicine and Health Management, Xiangya School of Public Health, Central South University, Changsha, Hunan, China; b Yale School of Public Health, New Haven, CT.

**Keywords:** adolescents, loneliness, reliability, UCLA loneliness scale, validity

## Abstract

Loneliness is prevalent and severe among adolescents, indicating the need for a reliable, valid, and concise instrument for detecting adolescent loneliness. This study aims to examine the psychometric properties of the Chinese version of the short-form UCLA Loneliness Scale (ULS-8) among Chinese adolescents.

Computer-assisted self-interviewing was used to complete the questionnaire among 3480 junior or senior high school students aged 10 to 19 years. Construct validity was assessed using exploratory factor analysis (EFA) and confirmatory factor analysis (CFA). To test the concurrent validity and convergent validity of the scale, a single loneliness item and variables such as depression, suicidal ideation, and quality of interpersonal relationships were used. For reliability, Cronbach alpha and test–retest correlation were computed.

Construct validity and internal consistency showed that the ULS-6, which excluded 2 reverse-scored items from the ULS-8, had stronger psychometric properties than the ULS-8. The convergent validity and concurrent validity were also supported by the study results. The overall Cronbach α of the ULS-6 was 0.878 and the test–retest reliability coefficient was 0.663.

The ULS-6 showed satisfactory reliability and validity in this study, suggesting that this instrument can be used in the measurement of loneliness among Chinese adolescents.

## 1 Introduction

Belongingness and social connection are among the fundamental needs of human beings. Loneliness can arise from a thwarted ability to meet these needs.^[1]^ Although loneliness is a pervasive experience across the lifespan, a substantial body of studies, including cross-sectional and cohort studies, have shown that loneliness tends to be more prevalent and more severe during adolescence.^[2,3]^ Globally, 12% to 26% of adolescents have reported moderate to high levels of loneliness.^[4,5]^ Longitudinal studies in the Netherlands, England, and Latino populations in the U.S. showed that 3% to 22% of adolescents experienced a persistent, high level of loneliness over 2 to 5 years.^[6–8]^ In China, regional data have shown that 21% to 33.4% of adolescents report a certain extended level of loneliness.^[9,10]^ Rich evidence suggests that adolescent loneliness is closely related to poorer mental health, including depression,^[11,12]^ anxiety,^[13,14]^ and suicidal behavior^[15–17]^; moreover, the impacts of adolescent loneliness can extend into adulthood. A longitudinal study using a nationally representative adolescent sample in the U.S showed that lonely adolescents not only more frequently reported depression across the period of adolescence but also showed increased diagnosed depression in early adulthood.^[18]^

There are 2 major types of self-report questionnaires for adolescent loneliness evaluation: multidimensional and unidimensional. Multidimensional questionnaires, such as the Louvain Loneliness Scale,^[19]^ Children Loneliness Scale,^[20]^ and Relational Provision Loneliness Questionnaire,^[21]^ are generally composed of subscales. These subscales assess not only loneliness but also conceptions closely related to loneliness, such as loneliness in different types of relationships (in parent–child relationships, in peer relationships, etc), satisfaction regarding social connections, competence in social interactions, feelings of aloneness, and social support.^[21]^

Unidimensional questionnaires offer the advantages of a more specific focus on loneliness and greater simplicity, compared with multidimensional questionnaires. These tools assess loneliness as a global experience shared by people who feel lonely and are generally single scale.^[22]^ The University of California Los Angeles (UCLA) Loneliness Scale (ULS) and its short versions (R-ULS, 1980; ULS-4, 1980; and ULS-8, 1987) are the most widely used unidimensional measures for adolescent loneliness globally as well as in China.^[23–25]^

The first version of the ULS, designed by Russell et al^[23]^ in 1978, included 20 items that were all negatively worded (such that an affirmative answer would suggest the presence of loneliness). In order to avoid systematic response bias, 10 items were reversed to be positively worded (reverse-scored) in the Revised UCLA version in 1980. The short versions ULS-8 and ULS-4 were developed on the basis of factor analysis of the revised UCLA-20. The study by Wilson et al^[26]^ among an adolescent sample showed that the revised UCLA-20 and ULS-8 have better reliability and validity than the ULS-4; the correlation coefficient between the revised UCLA-20 and ULS-8 was higher than between the revised UCLA-20 and ULS-4. Due to the advantage of its shorter length, ULS-8 is an ideal alternative to the revised UCLA-20.^[26]^ Despite the wide use of the ULS-8, to our knowledge, few studies have reported the psychometric properties of this instrument among mainland Chinese adolescents.

This study aims to examine the psychometric properties, in terms of reliability and validity, of a Chinese version of the ULS-8 among mainland Chinese adolescents.

## 2 Methods

### 2.1 Participants

Adolescents in this study were defined as students in junior or senior high school. Changsha city, located in the central south part of China, was the study site. Four schools in the city were randomly selected to represent the variety of types of schools by multistaged cluster random sampling, including 2 senior high schools (1 senior high school and 1 vocational high school) and 2 complete (junior and senior combined) high schools. The sampling unit was class, with 50 to 60 students in each class. All classes of first-year students and 15.4% of second-year students were randomly selected and recruited as study participants. Taking into account that third-year students in both junior and senior high school face more pressure in their studies due to exit examinations, they were not included in the study. On the basis of the school registration system, 3669 students were selected. Out of the 3669 students we approached, 44 (1.2%) students did not participate due to excused absence from school, 65 (1.8%) students failed to submit questionnaires due to technical problems, and 80 (2.2%) students refused to participate, leaving a sample of 3480 (94.8%) youths who completed the survey. Among these students, 1773 (50.9%) were male, and age ranged from 10 to 19 years (junior high school students aged 10–16 years, senior high school students aged 13–19 years). One thousand eighty-four (31.1%) were junior high school students and 2396 (68.9%) were senior high school students. From the 3480 students who completed the initial survey, 10% of participants were chosen by multistaged cluster sampling to take a retest within 4 weeks after the initial survey. The retest sampling unit was also class. Both first-year students and second-year students from these 4 schools were selected as the retest participants.

### 2.2 Procedure

The survey was incorporated into schools’ annual routine mental health surveys. The survey was administered as an electronic questionnaire and conducted during a computer course in each school in 2016. Before the survey, we selected 10 students from each of these 4 schools to conduct a pre-test and make sure that the scale was understood by all students. Other items in the questionnaire that were not part of the loneliness scale were also asked one by one among the students. According to the students’ feedback, the language of some items that were not part of the loneliness scale were adjusted to correct for ambiguous wording. Two trained investigators explained the survey, obtained informed consent from students before they took it, and were available to answer any questions throughout the survey. As the study was incorporated into schools’ routine mental health survey work, parents’ informed consent was obtained by the school ahead of the survey. Students who were identified during the course of the study as being in need of mental health services were carefully referred to professionals.

### 2.3 Instruments

#### 2.3.1 Chinese version of the ULS-8 Loneliness Scale

The ULS-8 Loneliness Scale^[25]^ contains 8 items, including 2 positively worded items (Item 3: “I am an outgoing person,” and Item 6: “I can find companionship when I want it”), which are reverse-scored. Each item has a 4-level frequency score, with answer choices of 1 (never), 2 (rarely), 3 (sometimes), and 4 (always). The total score ranges from 8 to 32 points, with higher scores suggesting a higher degree of loneliness. This study uses a Chinese version of the ULS-8 Loneliness Scale translated for a prior study conducted among Chinese participants by Zhou et al.^[27]^ Before the survey, we tested the scale among some of the subjects to see whether the items were consistent with the purpose of the test.

#### 2.3.2 Patient Health Questionnaire-9 (PHQ-9)

PHQ-9 is a self-assessment questionnaire with 9 items, assessing the depression symptoms of subjects over the 2 weeks before the time of the questionnaire.^[28]^ Each item is rated on a 4-point scale (0–3 points). The total score ranges from 0 to 27 points. A total score below 10 points indicates that the participant has no depressive symptoms, while a score of 10 or higher indicates the presence of depressive symptoms. The PHQ-9 Depression Scale has been translated into Chinese, and the reliability and validity among Chinese adolescent populations have been confirmed by prior studies.^[29]^

#### 2.3.3 Suicidal ideation items

According to the definition of suicidal ideation,^[30]^ we included an item (“Have you ever considered suicide seriously in the past 12 months?”) in the study questionnaire to assess suicidal ideation.

#### 2.3.4 Quality of relationships items

We incorporated 4 items to assess the quality of participants’ interpersonal relationships, including relationships with parents, leading teacher, and peers. We used 0 to 10 points to assess the quality of students’ relationships with parents and leading teacher, and 4 grades to judge the quality of relationships with classmates.

#### 2.3.5 The single loneliness item

In order to examine concurrent validity with the ULS-8, we also used the single loneliness item “I feel lonely” to assess loneliness directly, among the other questions that students answered on computer surveys. This item had a 4-level frequency score with answer choices of 1 (never), 2 (rarely), 3 (sometimes), and 4 (always).

### 2.4 Statistical analysis

Exploratory factor analysis (EFA) and confirmatory factor analysis (CFA) were used to test the construct validity of the Chinese version of the ULS-8. The sample was divided into 2 equal parts randomly, with one part used for EFA and one part used for CFA. Before EFA, a Kaiser–Meyer–Olkin (KMO) measure of sampling adequacy and Bartlett test of sphericity were conducted. The maximum likelihood was used for model estimation, as estimating parameters for non-normal categorical variables with the maximum likelihood method is generally acceptable if most variables have univariate skewness and kurtosis in the range −1.0 to +1.0.^[31,32]^ The goodness-of-fit index (GFI), normed fit index (NFI), comparative fit index (CFI), incremental fit index (IFI), adjusted goodness-of-fit index (AGFI), and root square error of approximation (RMSEA) were recorded for testing the fit of the model in CFA. The values of GFI, NFI, CFI, IFI, AGFI should be above 0.90 and RMSEA should be below the recommended level of 0.08.^[33]^ The correlation coefficient of the scale and single item “I feel lonely” were used to examine the concurrent validity. To test the convergent validity of the scale, variables such as depression, suicidal ideation, and quality of interpersonal relationships were adopted on the basis of existing literature.^[12,16,34]^

For reliability, the Cronbach alpha coefficient was computed to test the internal consistency reliability with α ≥0.70 as good internal consistency reliability.^[35]^ The test–retest reliability was tested based on the standard that the value should be extended to 0.60 when the time interval between the 2 measurements is more than 4 weeks.^[36]^ Data were analyzed with Statistical Product and Service Solutions 20.0 (SPSS 20.0) and Amos Graphics statistical software 21.0.

## 3 Results

### 3.1 Descriptive statistics of each item of the ULS-8

The descriptive statistics of each item of the ULS-8 are presented in Table 1. It includes mean, standard deviation, skewness, and kurtosis. (Details summarized in Table 1).

**Table 1:**
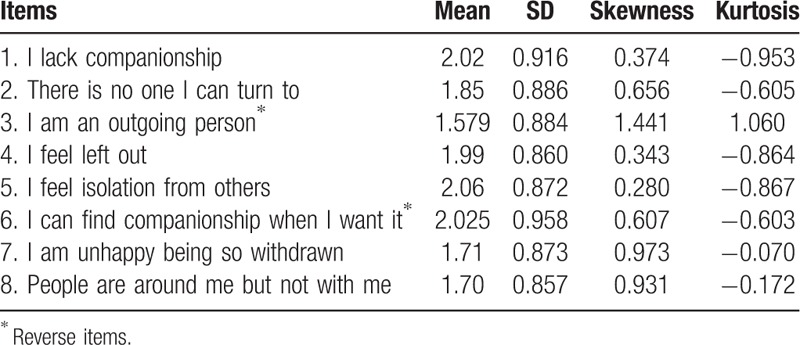
Descriptive statistics of each item of the ULS-8.

### 3.2 Construct validity

#### 3.2.1 Exploratory factor analysis (EFA) results

The size of the KMO measure (KMO = 0.850) and significance of Bartlett test (x^2^ = 5616.380, *P* < .001) revealed that the items of the ULS-8 had adequate common variance for factor analysis. Then, principal component analysis revealed 2 factors. Items 3 and 6, the 2 positively worded items, took place in the second factor and had a low contribution to the total variance (16.524%). Principal component analysis was conducted again after limiting the scale to a 1-factor structure based on the original design of the UCLA scale. The results revealed a 1-factor structure that explained 47.107% of the total variance (eigenvalues 2.94), with Items 3 and 6 having lower factor loadings (Table 2). Considering the results of principal component analyses and construction of the original scale, Items 3 and 6 were removed from the scale—resulting in a revised version of the scale with 6 items, the ULS-6.

**Table 2:**
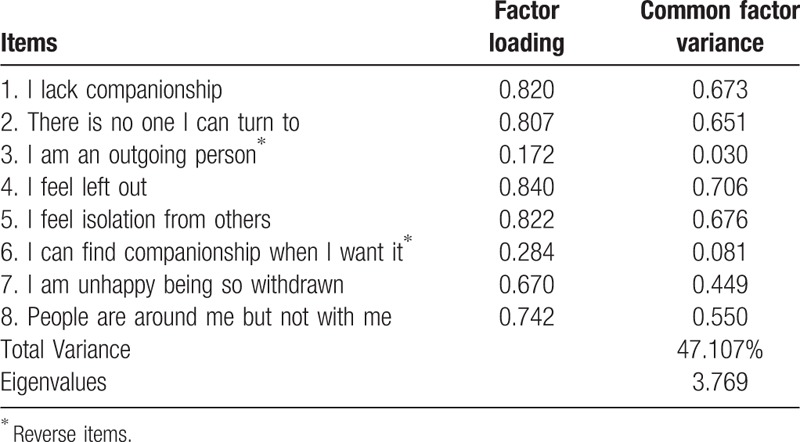
ULS-8 exploratory factor analysis and factor loading.

#### 3.2.2 Confirmatory factor analysis (CFA) results

**Figure 1:**
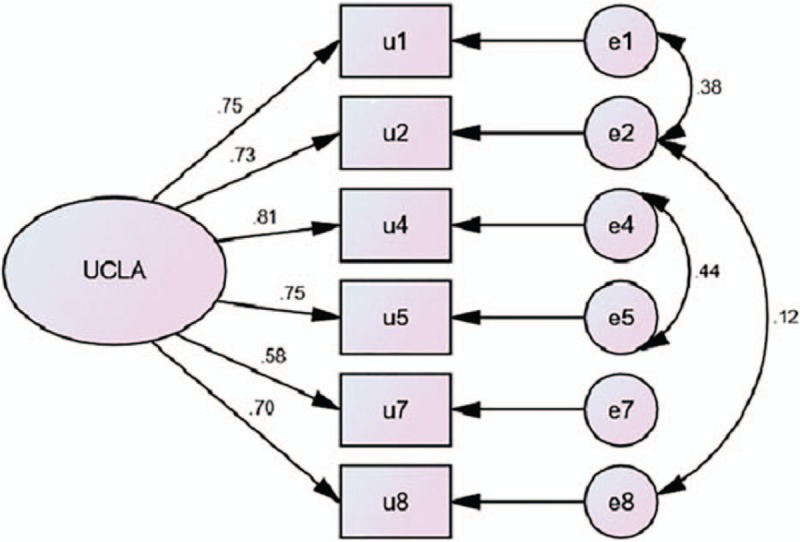
Path diagram of ULS-6.

We used the other half of the sample to conduct CFA on the 6-item 1-factor model. The results of CFA showed that the Chi-square value was 497.287(df = 9) with a high χ^2^/df (55.25), and the goodness of fit indices were GFI = 0.905, NFI = 0.908, CFI = 0.909, IFI = 0.909, AGFI = 0.778, and RMSEA = 0.177. These results indicated that the model should be adjusted, as the values of the first 5 indices should be above 0.90 and RMSEA should be below the recommended level of 0.08.^[33]^ According to the modification indices and theoretical knowledge, we established covariant relations between e1 and e2, e2, and e8, and e4 and e5 one after another to modify the model. After modifying the model, although the Chi-square test rejected the model [χ^2^(6) = 4.018, *P* < .05], the χ^2^/df was low. According to Wu,^[33]^ when the sample size is large, it is necessary to refer to other values to assess the whole model. The results of the goodness of fit indices were excellent (GFI = 0.996, NFI = 0.996, CFI = 0.997, IFI = 0.997, AGFI = 0.984, and RMSEA = 0.042). According to these results, the modified model had an excellent fit.^[33]^ The path diagram is presented in Fig. 1.

### 3.3 Concurrent validity

The correlation coefficient of the ULS-8 and single item “I feel lonely” was 0.713. The correlation between the ULS-6 and single item “I feel lonely” increased to 0.736 (*P* < .001).

### 3.4 Convergent validity

As Items 3 and 6 were deleted, we examined the convergent validity of ULS-6. To determine the convergent validity of ULS-6, we analyzed for the correlation between ULS-6 scores and depression; suicidal ideation; and quality of relationships with parents, leading teacher, and classmates. Table 3 presents the results of the correlation analyses among all variables. The ULS-6 was positively correlated with depression and suicidal ideation, and negatively correlated with the quality of relationships with mother, father, leading teacher, and classmates.

**Table 3:**
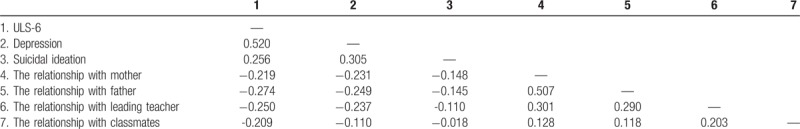
The correlation analyses among all variables.

### 3.5 Reliability

#### 3.5.1 Internal consistency

The Cronbach alpha coefficient, indicating consistency of the ULS-8, was α = 0.815. After deleting Item 3 (“I am an outgoing person”) and Item 6 (I can find companionship when I want it), the 2 reverse-scored items successively, the Cronbach alpha coefficients became 0.841 and 0.829, respectively. After removing both Item 3 and Item 6, the Cronbach alpha coefficients of the ULS-6 Loneliness Scale rose to 0.878. The correlation coefficient of each item score and total score of the ULS-8 Loneliness Scale was 0.355 to 0.788. The intercorrelations among the 8 items ranged from 0.035 to 0.771. After deleting both Item 3 and Item 6, the correlation coefficient of each item score and total score of the ULS-6 rose to 0.683 to 0.842. Also, the intercorrelations among the 6 items rose, ranging from 0.418 to 0.771.

#### 3.5.2 Test–retest reliability

Within 4 weeks, 352 participants took the survey again. The correlation between test and retest mean scores of these participants was 0.670. The test–retest reliability coefficient for the ULS-6 after deleting Item 3 and Item 6 was 0.663.

## 4 Discussion

The results of this study showed Item 3 and Item 6 (2 positive statement items) loaded in the second factor. After deleting these 2 items, the newly formed ULS-6 has good construct validity and convergent validity, high internal consistency, and acceptable test–retest reliability.

The original construction of the UCLA Loneliness Scale was unidimensional. In the revised UCLA scale, 10 items were changed from negative wording to positive wording to avoid systematic response bias.^[24]^ But this study as well as studies in other populations showed that the revised UCLA and UCLA Loneliness Scale (Version 3) had more than 1 factor; generally, negatively worded items all loaded in the same factor, and positively worded items loaded in the others.^[37,38]^ The results of this study also showed that the internal consistency and concurrent validity of ULS-6 (after deleting the 2 positively worded Items 3 and 6) were higher than those of the ULS-8, in a Chinese adolescent population. Similar results were reported by a study conducted among Chinese rural elderly that showed ULS-6, after deleting items 3 and 6, has better psychometric properties than ULS-8.^[27]^ Studies conducted among Turkish adolescents and college students in Taiwan also showed very low factor loading on Item 3 and Item 6.^[39,40]^ One possible explanation is that in a culture encouraging modesty such as China,^[41]^ positively worded items such as “I feel confident” are rated relatively lower on a self-report scale than the reality may be—a tendency that would have no impact on negatively worded items. This phenomenon could lead to discrepancy between positively worded and negatively worded items. However, it is still up for debate whether multifactors construction caused by wording only, or it is a multifactor scale. Another possible explanation could be that participants, especially younger adolescents, did not notice the difference in rating patterns between negatively and positively worded items. In this case, it would be best to highlight changes in rating patterns if keeping Items 3 and 6 in the scale for future studies.

Results of EFA showed that the ULS-6 had a single factor structure that was the same as the original scale, and was consistent with similar prior studies.^[27,39,40]^ The results of CFA showed that the ULS-6 model fit well with the data, and further verified the suitability of the structure, indicating that the scale had excellent structure validity. The close correlation with the single loneliness item also showed ULS-6 had good concurrent validity. On the basis of existing studies and theories of loneliness, adolescent loneliness usually has a slight negative association with quality of social relationships, social support, and social connectedness, and is moderately related to depression and suicide behavior.^[12,16,34]^ The correlations between ULS-6 and these variables were consistent with previous studies that indicated ULS-6 had good convergent validity in Chinese adolescent populations. And the Cronbach α of the ULS-6 in this study was credible based on the standard by Yu.^[35]^ This may suggest that the scale has good internal consistency and reliability. Although the test–retest reliability was slightly low (0.663), it was still acceptable, by the standards set forth by a study showing that the acceptable value of test–retest reliability should be extended to 0.60 when the time interval between the 2 measurements is more than 4 weeks.^[36]^

Several limitations should be mentioned in this study. First of all, we recruited all first-year and some second-year students from the selected schools, but did not enroll third-year students. Therefore, caution should be exercised when extrapolating results. Second-year students tend to experience a relatively higher degree of academic pressure, neither having time nor the corresponding computer courses to complete the survey. However, we recruited as many second-year students as possible to participate in the survey, and we believe that the results were representative of second-year students. Considering that third-year students are in a special period of study with unique pressures, future studies could specifically assess the mental health status of this population. In addition, this study was conducted in urban areas; therefore, future studies might examine applications of the ULS-6 among Chinese rural adolescents.

In conclusion, the results of this study indicate that the ULS-6 has good reliability and validity and is suitable for the measurement of loneliness among Chinese adolescents. The ULS-8 needs to be improved if it is to be used among Chinese adolescents in the future.

## Author contributions

**Conceptualization:** Shurong Xu, Mi Hu.

**Data curation:** Shurong Xu.

**Formal analysis:** Shurong Xu, Dan Qiu.

**Investigation:** Shurong Xu, Dan Qiu, Mei Zhao.

**Methodology:** Shurong Xu.

**Project administration:** Mi Hu.

**Validation:** Shurong Xu.

**Writing – original draft:** Shurong Xu.

**Writing – review & editing:** Jessica Hahne, Mi Hu.
